# Hematological malignancies in systemic lupus erythematosus: clinical characteristics, risk factors, and prognosis—a case-control study

**DOI:** 10.1186/s13075-021-02692-8

**Published:** 2022-01-03

**Authors:** Yuqi Zhang, Wei Li, Panpan Zhang, Jinyan Guo, Jinlei Sun, Jiameng Lu, Shengyun Liu

**Affiliations:** Department of Rheumatology and Immunology, the First Affiliated Hospital of Zhengzhou University, No.1 Jianshe East Road, Zhengzhou, 450052 Henan Province China

**Keywords:** Systemic lupus erythematosus, Hematological malignancies, Risk factors, Survival, Prognosis

## Abstract

**Background:**

Systemic lupus erythematosus (SLE) is a chronic and complex multi-system autoimmune disorder. Higher risks of hematological malignancies (HM) were observed in SLE patients, which was associated with higher mortality. The mechanism and risk factors of HM oncogenesis in SLE patients are still under investigation. The aim of this study was to explore clinical characteristics, risk factors, and prognosis of SLE patients with or without HM in the Chinese population.

**Methods:**

A retrospective, case-controlled study was conducted in 72 SLE patients between January 2013 and December 2020. Clinical and laboratory data were collected and compared between the two groups of patients with HM and those without HM. Logistic regression analysis was performed to determine risk factors of HM oncogenesis. The survival rate was estimated by Kaplan-Meier methods and Cox proportional hazards regression analysis.

**Results:**

Among 72 SLE patients in this study, fifteen complicated with HM and 57 without HM were identified. The incidence rate of HM was approximately 0.24% with elevated standardized incidence ratios of lymphoma and leukemia (27.559 and 12.708, respectively). Patients with HM were older when diagnosed with SLE, with a higher frequency of infection and splenomegaly, lower levels of hemoglobin and high-density lipoprotein compared with those without HM. Fewer patients with HM expressed positive anti-dsDNA antibody (26.7% vs 66.7%, *P* = 0.005) or received hydroxychloroquine treatment (40.0% vs 86.0%, *P* = 0.001). Older age at SLE diagnosis (*OR*=1.122, 95% *CI:* 1.037–1.214) was regarded as an independent risk factor of HM oncogenesis. Female (*RR=* 0.219, 95% *CI:* 0.070–0.681) and hydroxychloroquine (*RR=* 0.281, 95% *CI:* 0.094–0.845) were protective factors of mortality in SLE patients.

**Conclusions:**

SLE patients with an older age are at an increased risk of HM carcinogenesis. The prognosis of male patients with SLE tends to be poorer whether complicated with HM. The association of antinuclear antibody spectrum, medication, and HM oncogenesis in SLE needs further investigation.

**Supplementary Information:**

The online version contains supplementary material available at 10.1186/s13075-021-02692-8.

## Background

Systemic lupus erythematosus (SLE) is one of the common chronic and complex multi-system autoimmune disorders, which occurs predominantly in the reproductive age women, with the female-to-male ratio of approximately 10:1 [1, 2]. With early diagnosis and judicious therapy, including systemic glucocorticoids, immunosuppressive agents, and newly biological drugs, the survival rate of SLE has been significantly improved [3]. Study from a multisite international SLE cohort demonstrated a higher standardized mortality ratio compared to the general population, with particularly high mortality for circulatory disease, infection, renal disease, and malignancy [4]. Several studies have reported elevated cancer risks in SLE [5–14], especially hematological malignancies [11–21], which had an influence on the prognosis of patients [4, 11, 22]. While non-Hodgkin’s lymphoma (NHL) is the most common type, other kinds of hematological malignancies, such as Hodgkin’s lymphoma (HL), leukemia, and myeloma, are also at higher risks in SLE patients compared with the general population [14, 20]. However, the age-risk, gender-risk relationship and latency between hematological malignancies and SLE are still controversial [13, 18, 20, 23].

Hematological malignancies (HM) are a group of etiologically heterogeneous diseases. The association between SLE and HM is generally accepted to be due to intrinsic immunological dysregulation combined with exposure to medications and viruses [24–28]. However, elevated risks of treatment-induced malignancy remain controversial [5, 11, 15, 16, 29], partly because disease activity may influence the therapeutic choice. Therefore, it is a great challenge to attribute the occurrence of malignancies to the adverse effects of immunosuppressants [23, 30]. Up to now, most studies have focused on the incidence rate of various cancer kinds in SLE patients, while reports concerning clinical characteristics and outcome of SLE patients with HM are still limited, especially for Asians. Therefore, we conducted a retrospective study to investigate the clinical characteristics, laboratory parameters, risk factors and prognosis of Chinese SLE patients with HM.

## Methods

### Study design

We retrospectively collected all patients from our hospital with a diagnosis of SLE, as either a primary or a secondary diagnosis from January 2013 to December 2020. After excluding the repetitive cases, 7954 patients were identified. All patients met the American College of Rheumatology (ACR) or Systemic Lupus International Collaborating Clinics (SLICC) classification criteria [31, 32]. Exclusion criteria included patients younger than 18 or those previously diagnosed with other systemic rheumatoid diseases, such as rheumatoid arthritis, systemic sclerosis, inflammatory myopathy, and Sjogren’s syndrome. Hematological malignancies were identified by biological pathology of bone marrow or tissue specimens according to respective diagnostic criteria [33–35]. SLE patients diagnosed with HM synchronously were defined as HM diagnosis within a 2-month duration of SLE diagnosis. More than 2-month interval was defined as SLE prior or posterior to HM.

**Fig. 1:**
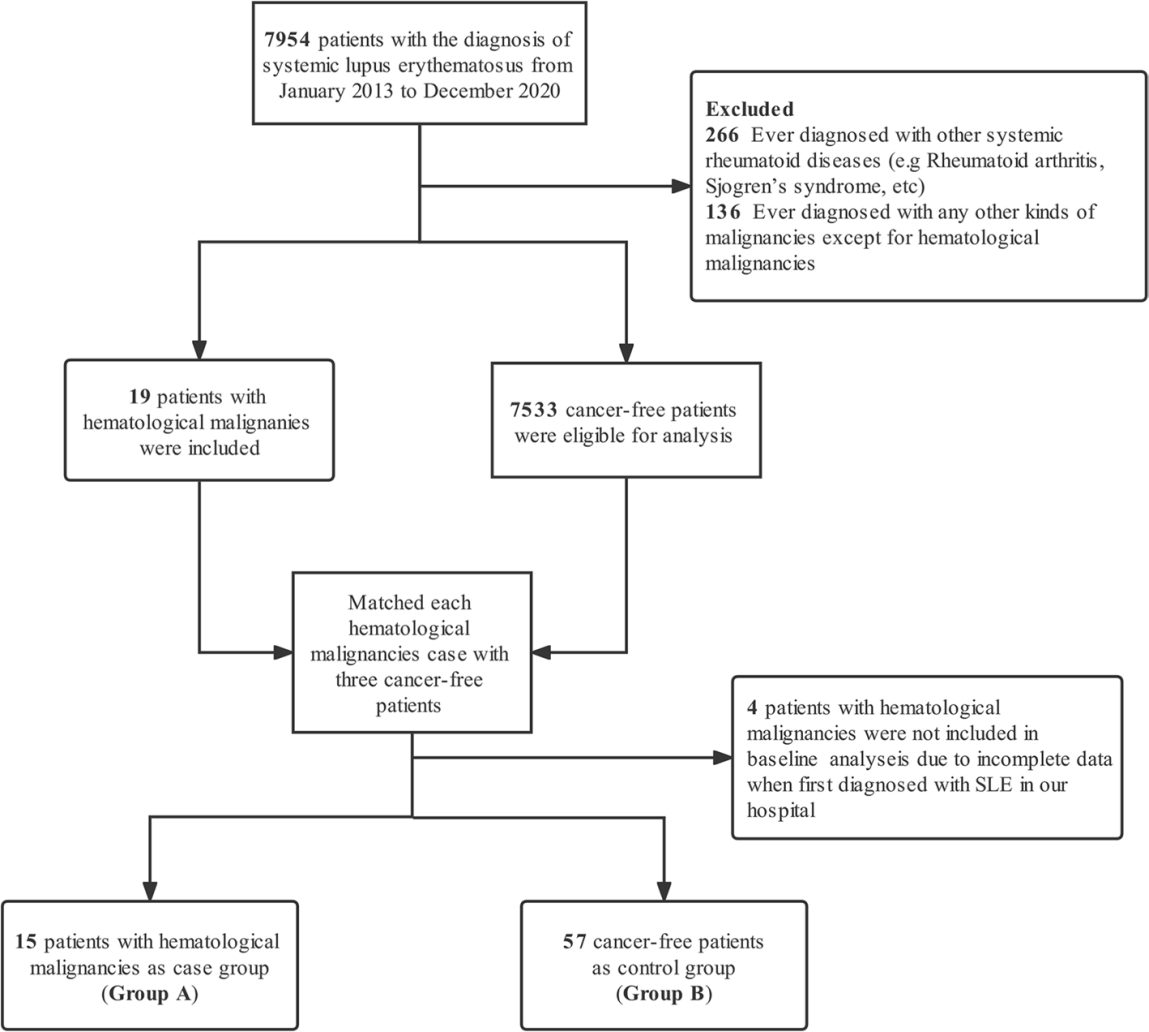
Flow chart of the study design

In total, 19 patients received a diagnosis of HM posterior to or synchronously with SLE, whereas four patients were excluded due to incomplete data at the baseline, leaving only 15 HM patients as the case group (Group A). In order to control confounding factors, 57 patients were randomly selected (table of random digits) from 7533 cancer-free patients as the control group (Group B) (Fig. 1). The study was approved by the Committee on scientific research and ethics of the First Affiliated Hospital of Zhengzhou University (No. 2019-KY-199).

### Clinical data and laboratory examinations

Patients’ data, including age, gender, medical history of chronic comorbidities, symptoms of onset, disease activity, treatment, outcome, and survival time, were collected. The follow-up was ended at the beginning of January 2021. The disease activity was evaluated according to systemic lupus erythematosus disease activity index-2000 (SLEDAI-2K) [36] at the diagnosis of SLE. Laboratory examinations including routine blood analysis, liver function, kidney function, C-reactive protein (CRP), erythrocyte sedimentation rate (ESR), complement, and autoantibody profile were collected when they were first diagnosed with SLE. Infection was defined if patients had clinical features of infection accompanied by sufficient laboratory data and imaging findings or microbiologically documented. From the date of SLE diagnosis to HM occurrence in Group A or the end of follow-up in Group B, all medications were retrieved from medical records and follow-up.

### Statistical analysis

Categorical variables are presented as frequencies (percentages), while continuous variables are reported as means with standard deviation (SD) or median with inter-quartile range (Q_1_–Q_3_). Independent-samples *t* tests or Mann-Whitney *U* tests were used to analyze normally or non-normally distributed data. Categorical data were analyzed using the Chi-square test or Fisher’s exact test. The data of sex- and age-stratified cancer incidence in the general Chinese population were published by the National Central Cancer Registry of China (NCCRC) [37]. The standardized incidence ratio (SIR) was calculated by dividing the observed malignant rate by the expected rate. Logistic regression analysis was performed to predict the risk factors. The survival rate was estimated by Kaplan-Meier methods and Cox proportional hazards regression analysis. A two-tailed *P* value < 0.05 was considered statistically significant. Statistical analyses were performed using IBM SPSS Statistics (version 25.0) and GraphPad Prism (version 8).

## Results

### Clinical characteristics

A total of 7954 patients with a diagnosis of SLE were identified, with 879 males (11.1%) and 7075 females (88.9%). Demographic and clinical characteristics are shown in Table 1. The groups were similar regarding gender, medical history of hypertension, diabetes mellitus, and dyslipidemia. However, patients in Group A were older than those in Group B when diagnosed with SLE [52 (42–63) vs 31 (25–47) years, *P* = 0.002]. None was reported a previous history of HM, while 10 (66.7%) patients developed HM synchronously with SLE and 5 (33.3%) posterior to SLE. Nine patients (60%) in Group A had fever at the time of admission, with no significant difference between Groups A and B. Nine patients (60%) in Group A suffered from infection at the time of SLE diagnosis, demonstrating a higher frequency than Group B (22.8%). All the infections in Group A were pneumonia, and no significant predominance was shown in pathogens, including *Klebsiella pneumonia* (2, 22.2%), *Cytomegalovirus* (1, 11.1%), *Pneumocystis jirovecii* (1, 11.1%), and unidentified microorganisms (5, 55.6%). Pneumonia was the most common infection in Group B (11/13, 84.6%), while the other two cases were panniculitis and upper respiratory infection, respectively. The reported pathogens in Group B were *Klebsiella pneumonia* (2, 15.4%), *Streptococcus pneumonia* (2, 15.4%), *Pneumocystis jirovecii* (1, 7.7%), *Mycobacterium tuberculosis* (1, 7.7%), and *Cytomegalovirus* (1, 7.7%). Common SLE symptoms such as rash, arthritis, and oral ulceration were similar between the two groups, but more patients in Group A had splenomegaly than those in Group B (46.7% vs. 15.8%, *P* = 0.027).

**Table 1:** Clinical features of SLE patients with/without hematological malignancies

Parameter	Group A (***n***=15)	Group B (***n***=57)	***P*** value
Female (*n*, %)	12 (80.0)	49 (86.0)	0.867
Age at SLE diagnosis (years), M (Q1–Q3)	52 (42–63)	31 (25–47)	0.002*
Age at HM diagnosis (years, mean ± SD)	52±15	-	-
Lymphadenopathy (*n*, %)	9 (60.0)	26 (45.6)	0.321
Fever (*n*, %)	9 (60.0)	21 (36.8)	0.106
Infection (*n*, %)	9 (60.0)	13 (22.8)	0.014*
Hypertension (*n*, %)	1 (6.7)	9 (15.8)	0.625
Diabetes mellitus (*n*, %)	0 (0)	1 (1.8)	1.000
Dyslipidemia (*n*,%)	13 (13/14, 92.9)	40 (40/56, 71.4)	0.186
Smoking (*n*, %)	1 (6.7)	5 (8.8)	1.000
Alcohol consumption (*n*, %)	1 (6.7)	2 (3.5)	1.000
Family history of tumor (*n*, %)	2 (13.3)	4 (7.0)	0.793
Rash (*n*, %)	4 (26.7)	20 (35.1)	0.538
Arthralgia (*n*, %)	5 (33.3)	14 (24.6)	0.721
Pleural effusion (*n*, %)	5 (33.3)	15 (26.3)	0.829
Pericardial effusion (*n*, %)	5 (33.3)	16 (29.1)	0.936
Splenomegaly (*n*, %)	7 (46.7)	9 (15.8)	0.027*

*Statistical significance (*P*<0.05). Group A: SLE patients with hematological malignancies; Group B: SLE patients without hematological malignancies

*Abbreviation*: *SLE* systemic lupus erythematosus, *HM* hematological malignancies

### Laboratory findings

Laboratory parameters of 72 SLE patients were elaborated in Table 2. Majority of patients in both groups suffered from hematological abnormality (86.7% vs 68.4%, *P* = 0.280), including leukocytopenia, thrombocytopenia, or anemia. Patients in Group A had lower levels of hemoglobin, high-density lipoprotein, and total cholesterol than those in Group B, whereas C-reactive protein and ferritin were significantly higher in Group A compared with Group B. As for the expression of autoantibodies, anti-nuclear antibody presented positively in all the patients at the baseline. Fewer patients in Group A expressed anti-dsDNA antibody (26.7% vs 66.7%, *P* = 0.005), whereas other kinds of antibodies were comparably expressed in the two groups. For five patients in Group A and 22 in Group B with available lymphocyte subset data, the ratio of CD4+/CD8+ T cell [0.54 (0.48–0.79) vs 0.92 (0.57–1.34), *P* = 0.080] and percentage of B cell (9.06±7.43 vs 19.49±14.45, *P* = 0.137, Additional file 1) seemed to be lower in Group A, but it still need further confirmation. Disease activity estimated by SLEDAI-2K at the diagnosis of SLE did not show a significant difference between the two groups [9.00 (4.00–14.00) vs 12.00 (8.00–18.00), *P* = 0.184].

**Table 2:** Laboratory parameters of SLE patients with/without HM at the baseline

Parameters	Group A (***n***=15)	Group B (***n***=57)	***P*** value
Hematological abnormality (*n*, %)	13 (86.7)	39 (68.4)	0.280
WBC (×10^9^), M (Q1-Q3)	3.40 (1.80–5.68)	3.90 (2.85–6.25)	0.072
RBC (×10^12^, mean ± SD)	3.08±0.88	3.59±0.64	0.016*
Hb (g/L, mean ± SD)	89.97±23.18	103.43±20.41	0.031*
Platelet (×10^9^, mean ± SD)	118.80±64.68	160.54±82.66	0.074
Urine protein positivity (*n*, %)	5 (33.3)	27 (47.4)	0.330
24hTP (g), M (Q1–Q3)^a^	0.49 (0.29–1.13)	1.40 (0.36–4.92)	0.156
Scr (umol/L), M (Q1–Q3)	62.00 (47.50–73.50)	55.00 (47.50–73.50)	0.856
GFR (ml/min/1.73m^2^), M (Q1–Q3)	85.57 (77.04–109.31)	108.76 (91.70–121.71)	0.099
TP (g/L, mean ± SD)	66.88±11.74	64.72±13.16	0.567
Albumin (g/L, mean ± SD)	29.70±6.69	31.72±7.58	0.351
Globin (g/L, mean ± SD)	37.18±13.39	33.33±9.64	0.310
Lupus nephritis (*n*, %)	5 (33.3)	28 (49.1)	0.275
TC (mmol/L), M (Q1–Q3)	3.05 (2.54–3.45)	3.91 (3.09–4.56)	0.030*
TG (mmol/L), M (Q1–Q3)	1.33 (0.75–2.02)	1.64 (1.20–2.37)	0.157
HDL (U/L), M (Q1–Q3)	0.61 (0.42–0.69)	0.96 (0.77–1.16)	0.003*
LDL (U/L), M (Q1–Q3)	1.74 (1.11–2.34)	2.39 (1.67–2.95)	0.058
ALT (U/L), M (Q1–Q3)	24.00 (10.00–35.00)	23.00 (13.00–49.00)	0.682
AST (U/L), M (Q1–Q3)	29.00 (17.00–58.00)	28.00 (18.00–56.50)	0.950
LDH(U/L), M (Q1–Q3)	262.00 (197.00–453.00)	273.00 (206.00–614.00)	0.767
EBV-IgM+ (*n*, %)	0 (0/7, 0)	2 (2/20, 10.0)	0.975
EBV-IgG+ (*n*, %)	7 (7/7, 100)	20 (20/20, 100)	-
C3 (g/L, mean ± SD)	0.78±0.35	0.67±0.36	0.314
C4 (g/L, mean ± SD)	0.16±0.12	0.13±0.08	0.401
ESR (mm/h), M (Q1–Q3)	48.00 (34.50–100.25)	41.50 (19.75–74.00)	0.533
CRP (mg/L), M (Q1–Q3)	16.31 (2.82–34.71)	3.88 (1.50–7.17)	0.018*
Ferritin (ng/ml), M (Q1–Q3) ^b^	692.45(475.20–1248.28)	188.85 (71.92–369.53)	0.007*
SLEDAI-2K, M (Q1–Q3)	9.00 (4.00–14.00)	12.00 (8.00–18.00)	0.184
ANA (*n*, %)	15 (100)	57 (100)	-
Anti-dsDNA antibody (*n*, %)	4 (26.7)	38 (66.7)	0.005*
Anti-Smith antibody (*n*, %)	5 (5/11, 45.5)	19 (19/50, 38.0)	0.907
Anti-Rib P (*n*, %)	4 (4/14, 28.6)	26 (26/54, 48.1)	0.189
Anti-Nuc (*n*, %)	5 (5/12, 41.7)	31 (31/55, 56.4)	0.355
Anti-His (*n*, %)	3 (3/13, 23.1)	25 (25/54, 46.3)	0.128
Anti-SSA/Ro52 (*n*, %)	9 (9/13, 69.2)	24 (24/52, 46.2)	0.137
Anti-SSA/Ro60 (*n*, %)	8 (8/12, 66.7)	26 (26/49, 53.1)	0.395
Anti-SSB (*n*, %)	4 (4/12, 33.3)	9 (9/53, 17.0)	0.379
ACA (*n*, %)	2 (2/8, 25.0)	2 (2/49, 4.1)	0.161
APL (*n*, %)	4 (4/7, 57.1)	15 (15/27, 55.6)	1.000

* Statistical significance (*P*<0.05)

Group A: SLE patients with hematological malignancies; Group B: SLE patients without hematological malignancies

Hematological abnormality: Patients present with leukopenia (WBC<3.5×10^9^), anemia (Hb<120g/L for male or Hb<110g/L for female), or thrombocytopenia (platelet<100×10^9^)

Urine protein positivity: 24-h total urinary protein >0.15g or spot urine protein/creatinine ratio>200mg/g or positive results in qualitative test of urinary protein

Lupus nephritis: 24-h total urinary protein ≥ 0.5g or the confirmation of renal biopsy

^a^ The data of Group A (7 patients) and Group B (29 patients)

^b^ The data of Group A (6 patients) and Group B (20 patients)

*Abbreviations*: *SLE* systemic lupus erythematosus, *HM* hematological malignancies, *WBC* white blood cell, *RBC* red blood cell, *Hb* hemoglobin, *24hTP* 24-h total urinary protein, *Scr* serum creatinine, *GFR* glomerular filtration rate, *TP* total protein, *TC* total cholesterol, *TG* triglyceride, *HDL* high-density lipoprotein, *LDL* low-density lipoprotein, *ALT* alanine transaminase, *AST* aspartate transaminase, *LDH* lactic dehydrogenase, *EBV* Epstein-Barr virus, *CMV* cytomegalovirus, *ESR* erythrocyte sedimentation rate, *CRP* C-reactive protein, *SLEDAI-2K* systemic lupus erythematosus disease activity index-2000, *ANA* anti-nuclear antibody, *Anti-Rib P* anti-ribosomal P-protein antibody, *Anti-Nuc* anti-nucleosome antibody, *Anti-His* anti-histone antibody, *ACA* anti-centromere antibody, *APL* anti-phospholipid antibody

### Types of hematological malignancies and SIR

Different HM types in Group A were elaborated (Additional file 2). Among 15 cases with HM, ten patients developed HM synchronously with SLE while five posterior to SLE. Seven patients (46.7%) developed NHL, and the most frequently observed diagnosis was diffuse large B cell lymphoma (DLBCL, 26.7%), followed by acute myeloid leukemia (AML) in four patients (26.7%). The remaining four cases contained two HL (13.3%) and two multiple myelomas (MM, 13.3%).

Among 7954 SLE patients admitted to our hospital from 2013 to 2020, in total of nineteen patients were diagnosed with HM (Additional file 2), with the incidence rate of approximately 0.24%. There was a significantly increased SIR of lymphoma (27.559, 95% *CI*: 10.437–72.766) and leukemia (12.708, 95% *CI*: 4.086–39.524). Among the 19 patients with HM, three were male (3/879, 0.34%), while 16 were female (16/7075, 0.23%), inferring gender difference of HM incidence in SLE patients.

### Risk factors of hematological malignancies in SLE patients

Based on the baseline comparisons above, risk factors estimated by logistic regression analysis are displayed in Table 3. The univariate logistic analysis revealed that older age at SLE diagnosis (*OR*=1.075, 95% *CI:* 1.028–1.125), splenomegaly (*OR*=4.667, 95% *CI*: 1.351–16.115), and infection (*OR*=5.077, 95% *CI:* 1.523–16.925) were associated with high HM risk, whereas hemoglobin (*OR*=0.970, 95% *CI:* 0.945–0.998), high-density lipoprotein (*OR*=0.029, 95% *CI*: 0.002–0.359), and anti-dsDNA antibody (*OR*=0.182, 95% *CI:* 0.051–0.647) were considered as protective factors for HM risk. In multivariate analysis, only older age at SLE diagnosis (*OR*=1.122, 95% *CI*: 1.037-1.214) was regarded as a risk factor.

**Table 3:** Risk factors of hematological malignancies development in SLE patients

Parameter	Univariate logistic analysis		Multivariate logistic analysis	
	***OR***(95% ***CI***)	***P*** value	***OR***(95% ***CI***)	***P*** value
Age at SLE diagnosis	1.075 (1.028–1.125)	0.002*	1.122 (1.037–1.214)	0.004*
Infection	5.077 (1.523–16.925)	0.008*	4.289 (0.598–30.768)	0.148
Splenomegaly	4.667 (1.351–16.115)	0.015*	2.051 (0.177–23.739)	0.565
Hemoglobin	0.970 (0.945–0.998)	0.038*	0.959 (0.918–1.001)	0.055
TC	0.499 (0.244–1.022)	0.057	-	-
HDL	0.029 (0.002–0.359)	0.006*	0.039 (0.001–1.383)	0.075
CRP	1.011 (0.990–1.033)	0.320	-	-
Anti-dsDNA antibody	0.182 (0.051–0.647)	0.009*	0.238 (0.035–1.636)	0.145
SLEDAI-2K at SLE diagnosis	0.952 (0.873–1.038)	0.264	-	-

*Statistical significance (*P*<0.05)

*Abbreviations*: *SLE*, systemic lupus erythematosus; *TC*, total cholesterol; *HDL*, high-density lipoprotein; *CRP*, C-reactive protein; *SLEDAI-2K*, systemic lupus erythematosus disease activity index-2000

### Treatment and prognosis for SLE patients

Treatment strategies were displayed in Table 4. Thirteen (86.7%) patients in Group A and fifty-four (94.7%) in Group B received glucocorticoids, with no statistical significance. No one was exposed to cyclophosphamide (CYC) prior to the diagnosis of HM in Group A whereas 9 patients (15.8%) in Group B received CYC treatment. There was no significant difference between the two groups regarding drug exposure, except for hydroxychloroquine (HCQ). Fewer patients in Group A (40.0%) were treated with HCQ than Group B (86.0%). Besides, HCQ was regarded as a protective factor for HM oncogenesis in SLE patients (*OR*=0.143, 95% *CI*: 0.041–0.504) by univariate logistic regression analysis (Additional file 3). Concerning the small proportion of drug exposure, further multivariate analysis was not performed.

**Table 4:** Treatment for SLE with/without hematological malignancies

Medication	Group A (***n***=15)	Group B (***n***=57)	***P*** value
Glucocorticoids (*n*, %)	13 (86.7)	54 (94.7)	0.601
Pulse therapy of glucocorticoids (*n*, %)	2 (13.3)	7 (12.3)	1.000
IVIG (*n*, %)	3 (20.0)	17 (29.8)	0.666
Cyclophosphamide (*n*, %)	0 (0)	9 (15.8)	0.228
Mycophenolate mofetil (*n*, %)	2 (13.3)	19 (33.3)	0.231
Methotrexate (*n*, %)	0 (0)	2 (3.5)	1.000
Leflunomide (*n*, %)	0 (0)	5 (8.8)	0.536
Azathioprine (*n*, %)	1 (6.7)	2 (3.5)	1.000
Cyclosporin (*n*, %)	0 (0)	2 (3.5)	1.000
Tacrolimus (*n*, %)	1 (6.7)	6 (10.5)	1.000
Thalidomide (*n*, %)	2 (10.5)	1 (1.8)	0.204
Hydroxychloroquine (*n*, %)	6 (40.0)	49 (86.0)	0.001*
Rituximab (*n*, %)	0 (0)	3 (5.3)	0.856
Belimumab (*n*, %)	0 (0)	1 (1.8)	1.000

*Statistical significance (*P*<0.05). Group A: SLE with hematological malignancies; Group B: SLE without hematological malignancies.

*Abbreviation*: *IVIG* intravenous immunoglobin

**Fig. 2:**
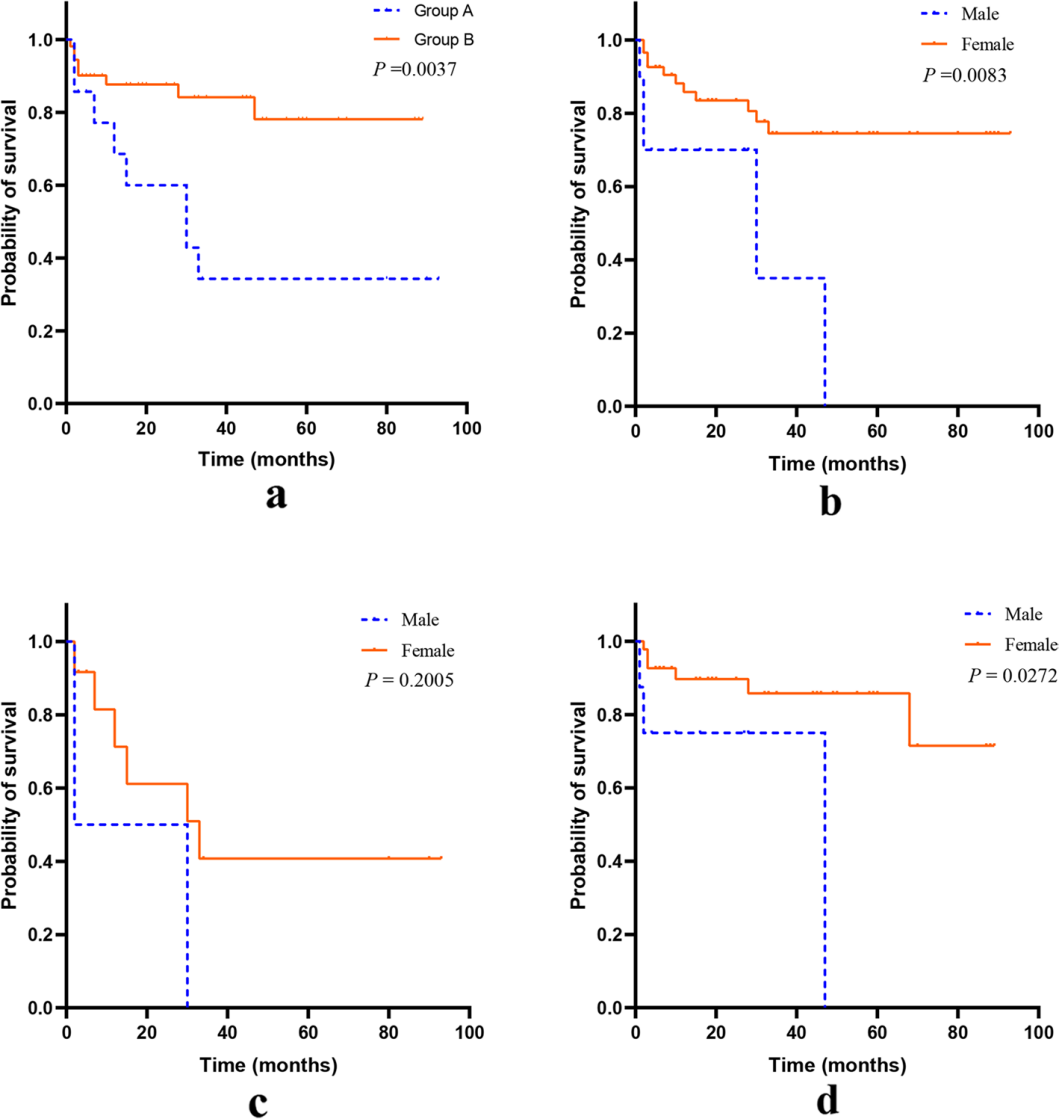
Kaplan-Meier curves comparing patients’ survival. **a** Between SLE patients with and without HM. **b** Between male and female in 72 SLE patients. **c** Between male and female in SLE patients with HM. **d** Between male and female in SLE patients without HM. Abbreviations: SLE, systemic lupus erythematosus; HM, hematological malignancies.

**Fig. 3:**
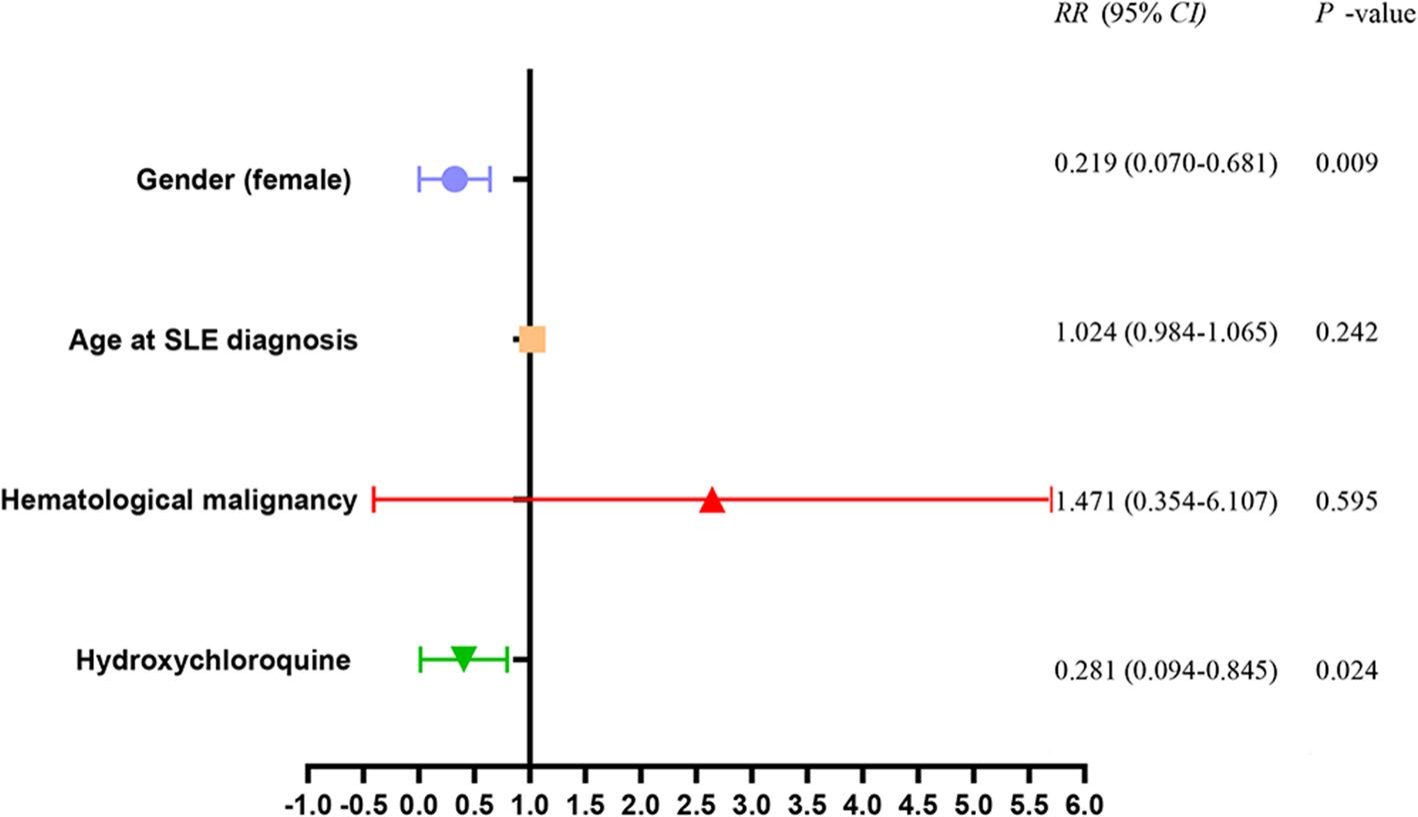
Multivariate proportional hazards Cox regression on risk factors of overall mortality in SLE patients. Abbreviation: SLE, systemic lupus erythematosus

The prognosis estimated by Kaplan-Meier methods was shown in Fig. 2. Up to January 2021, the median follow-up period was 22.5 months and the median survival time for Group A was 30 months from SLE diagnosis and 15 months from HM diagnosis. Patients in Group B had a significantly better prognosis than those in Group A (*P* = 0.0037, Fig. 2a). Male tended to have a worse prognosis no matter if they were complicated with HM (Fig. 2b–d). The risk factors of overall mortality estimated by Cox regression (Fig. 3) showed that female (*RR=* 0.219, 95% *CI:* 0.070–0.681) and hydroxychloroquine (*RR=* 0.281, 95% *CI:* 0.094–0.845) were regarded as protective factors. The main cause of death for all the patients was multiple organ failure due to malignancy or pulmonary infection.

## Discussion

Numerous studies have reported an increased risk of HM in SLE patients [16, 19, 20], and the mechanism between HM and SLE remains under exploration. SLE is characterized by immune dysregulation with lymphocyte hyperactivity [16]. Intrinsic immunological dysregulation combined with external exposure to medications and viruses may contribute to HM carcinogenesis in SLE patients [25, 26]. However, there is still a lack of sufficient evidence to support the contribution of infection and medication to HM risk in SLE patients. Besides, studies concerning clinical characteristics and outcomes of SLE patients with HM are still limited. Therefore, we conducted a retrospective, case-control study to elaborate clinical characteristics, laboratory parameters, and prognosis of SLE patients complicated with HM in our hospital. In addition, risk factors for HM oncogenesis in SLE patients were also identified.

Significant increased SIRs of leukemia and lymphoma were proved in SLE patients in our study compared with reported incidence of HM in general Chinese population [37]. NHL was the most common type as other studies [6, 38] and DLBCL, a relatively aggressive type, accounted for more than half of NHL in our study. Most of the patients in our study were diagnosed with HM within two-month latency of SLE, which was confirmed in previous cohort studies [6, 13] that the risk ratio of hematological cancers decreased with time, with the highest risk in the first year after SLE diagnosis. Malignant B cells may produce immunoglobulin with autoantibody features in NHL [11]. Besides, the symptoms of lymphoma may mimic SLE, like fever, arthralgia, and lymphadenopathy, so if the symptoms are unusually severe or persistent, biopsy of bone marrow or lymph nodes is necessary to clarify if a malignancy has occurred.

The prevalence of HM and overall mortality was higher in male patients compared with female, in accord with previous studies [13, 23, 38, 39], so males may need more vigilance during the follow-up. Older age at the time of SLE diagnosis was an independent risk factor in our study, in accord with some previous studies [5, 40] but in conflict with a Taiwanese study that younger patients had a greater risk ratio of cancer [13]. However, for SLE patients complicated with HM, patients between 40 and 69 years had the highest SIR [13], which was comparable with our patients. Our study indicated that more SLE patients with HM suffered from infection at the time of SLE diagnosis. Due to impaired cellular and humoral immune functions, SLE patients are susceptible to infection which would be induced by bacteria, viruses, or parasites [41]. However, studies demonstrating the role of infection in SLE and HM remain scarce. Johnson et al. demonstrated that SLE patients had increased clinically relevant Epstein-Barr virus (EBV) infection, which was associated with risk of hematological cancers [42]. However, the relationship among infection microorganism, SLE, and tumorigenesis still need further research, and infection prevention may be necessary for SLE patients to decrease their HM risk.

Patients with HM had lower hemoglobin in our study, while a nested case-control study also indicated that hematologic aberrations (leukocytopenia/thrombocytopenia or hematologic anemia) were associated with NHL in SLE patients [29]. Anemia may be caused by a variety of conditions in SLE, with hemolytic anemia as a common feature. Hemminki et al [21] concluded that autoimmune hemolytic anemia was correlated with increased risk of lymphoma and leukemia. However, non-hemolytic anemia associated with HM needs more investigation. Cardiovascular disease is one of the major causes of morbidity and mortality in patients with SLE [4, 43]. As one of the traditional risk factors of cardiovascular disease, dyslipidemia was common in SLE patients, and it was correlated with disease activity [44, 45]. Dyslipidemia usually refers to elevated total cholesterol, triglycerides, low-density lipoprotein, and decreased high-density lipoprotein levels. However, the influence of lipid parameters on cancer is still unclear [46]. In our study, high-density lipoprotein was lower in patients with HM and was one of the protective factors of HM oncogenesis, which still required further investigation.

Antinuclear antibodies (ANAs) are a spectrum of autoantibodies that react with various nuclear and cytoplasmic components of cells. ANAs may have anti-tumor activity and could be mediated by antibody-dependent cell-mediated cytotoxicity [47]. Lü et al. [48] illustrated that anti-dsDNA antibodies have an inhibitory effect on tumor cells via inducing apoptosis. Hansen et al. [49] demonstrated that anti-DNA antibodies might have direct anti-cancer effects in cells with DNA repair defects. Our study showed SLE patients with HM have a lower positive rate of anti-dsDNA and in univariate logistic analysis, anti-dsDNA showed a protective effect on HM carcinogenesis. However, the association between ANAs and risk of HM oncogenesis is still inconclusive and needs further confirmation.

The influence of medications remains debatable. Some studies suggested an increased risk of lymphoma associated with corticosteroids, especially high cumulative steroids [23], probably due to high disease activity or severity of the underlying disease [50], so it may be too arbitrary to draw a conclusion between steroid treatment and lymphoma. Hsu et al. [51] illustrated that higher cumulative CYC dose and lower HCQ dose were associated with higher cancer risks. Ertz-Archambault et al. [52] found azathioprine exposure was associated with a 7-fold risk for myeloid neoplasm. Several studies launched the hypothesis of a protective action of antimalarials like HCQ against cancer in patients with SLE [9, 53]. Besides, a prospective SLE cohort study demonstrated that the risk of mortality in the HCQ group was lower than that in the control group (hazard ratio = 0.68, 95% *CI*: 0.56–0.82), indicating the survival protective effect of HCQ adherence [54]. In our study, fewer SLE patients with HM received HCQ and HCQ showed a protective effect on decreasing risks of mortality in the analysis. However, majority of patients with HM were diagnosed synchronously with SLE, so the treatment strategy of SLE might be affected resulting in vacancy of HCQ consumption.

Our study has several limitations. First, it’s a retrospective, single-center study from a relatively small cohort in China, so additional multi-center, prospective research is required. Second, infection history, and classification of pathogens should be further investigated, which may be associated with HM oncogenesis. Third, cumulative dosages of immunosuppressants were lacking, which need further exploration in the future. However, we have done a detailed analysis between SLE patients with and without HM about clinical characteristics, risk factors, and outcomes in our hospital, which may remind more clinicians to be concerned about HM during SLE follow-up.

## Conclusions

SLE patients have an increased risk of developing with hematological malignancies than the general population, especially for those at a higher age when diagnosed with SLE. The prognosis of male patients with SLE tends to be poorer whether complicated with HM. The protective role of hydroxychloroquine in HM occurrence and mortality of SLE patients, and the association of autoantibodies with HM oncogenesis still need further confirmation.

## Supplementary Information

**Additional file 1: Supplementary Table 1**. The lymphocyte subsets of SLE with/without hematological malignancies.

**Additional file 2: Supplementary Table 2**. Clinical features of 19 SLE patients with hematological malignancies.

**Additional file 3: Supplementary Table 3**. The effects of medication exposure on HM risk in SLE patients.

## Data Availability

The datasets used and/or analyzed during the current study are available from the corresponding author on reasonable request.

## Abbreviations

ANAs: Antinuclear antibodies
AML: Acute myeloid leukemia
CYC: Cyclophosphamide
DLBCL: Diffuse large B cell lymphoma
EBV: Epstein-Barr virus
HCQ: Hydroxychloroquine
HL: Hodgkin’s lymphoma
HM: Hematological malignancies
MM: Multiple myeloma
NCCRC: National Central Cancer Registry of China
NHL: Non-Hodgkin’s lymphoma
OR: Odds ratio
RA: Rheumatoid arthritis
RR: Risk ratio
SD: Standard deviation
SIR: Standardized incidence ratio
SLE: Systemic lupus erythematosus
SLEDAI-2K: Systemic lupus erythematosus disease activity index-2000
SS: Sjogren’s syndrome
