# Supplementary material for: Hematological malignancies in systemic lupus erythematosus: clinical characteristics, risk factors, and prognosis—a case-control study
Source: Arthritis Res Ther. 2022 Jan 3;24:5. doi: 10.1186/s13075-021-02692-8 (PMC8722144; doi:10.1186/s13075-021-02692-8)
Supplement: Supplementary file 1 — Additional file 1: Supplementary Table 1. The lymphocyte subsets of SLE with/without hematological malignancies. [file 13075_2021_2692_MOESM1_ESM.pdf]

Supplementary Table 1. The lymphocyte subsets of SLE with/without hematological malignancies

**Lymphocyte subsets (%)**	**Group A (n=5)**	**Group B (n=22)**	***P*-value**
CD3+ T cell	68.90±17.56	70.48±13.42	0.735
CD3+CD8+ T cell	39.26±7.97	34.87±12.27	0.546
CD3+CD4+ T cell	24.48±11.04	34.72±15.30	0.164
CD4+/CD8+ T cell (M, Q1-Q3)	0.54 (0.48-0.79)	0.92 (0.57-1.34)	0.080
NK cell (CD16+CD56+)	21.38±21.72	12.62±9.75	0.425
B cell (CD19+)	9.06±7.43	19.49±14.45	0.137

The data of lymphocyte subsets were from 5 patients in Group A and 22 patients in Group B. Group A: SLE patients with hematological malignancies; Group B: SLE patients without hematological malignancies.
