# Supplementary material for: Hematological malignancies in systemic lupus erythematosus: clinical characteristics, risk factors, and prognosis—a case-control study
Source: Arthritis Res Ther. 2022 Jan 3;24:5. doi: 10.1186/s13075-021-02692-8 (PMC8722144; doi:10.1186/s13075-021-02692-8)
Supplement: Supplementary file 2 — Additional file 2: Supplementary Table 2. Clinical features of 19 SLE patients with hematological malignancies. [file 13075_2021_2692_MOESM2_ESM.pdf]

Supplementary Table 2. Clinical features of 19 SLE patients with hematological malignancies

**Patient**	**Sex**	**Age at SLE diagnosis (y)**	**Age at HM diagnosis (y)**	**Latency between HM and SLE (months)**	**SLEDAI-2K at diagnosis of SLE**	**SLEDAI-2K at diagnosis of HM**	**Type of HM (Grade)**	**Biopsy site to confirm HM**	**Main treatment to HM**	**Outcome (To the end of follow-up period)**
P1	F	46	46	Synchronously	3	3	AML-M2	Bone marrow	Chemotherapy	Survival
P2	M	27	27	Synchronously	4	4	AML-M2	Bone marrow	Chemotherapy	Not applicable
P3	F	54	54	Synchronously	9	9	MM	Bone marrow	Chemotherapy +ASCT	Survival
P4	F	63	63	Synchronously	10	10	NHL-DLBCL(IV)	Antrum of stomach	Chemotherapy	Dead
P5	M	43	43	Synchronously	4	4	AML-M2	Bone marrow	Chemotherapy	Dead
P6	F	63	63	Synchronously	24	24	HL-Mixed cellularity(IV)	Lymph node	Chemotherapy	Dead
P7	M	75	77	18	4	18	NHL-DLBCL(IV)	Head skull	Chemotherapy	Dead
P8	F	23	24	16	23	18	AML-M3	Bone marrow	Chemotherapy	Survival
P9	F	42	48	75	12	5	NHL-MALT(III)	Parotid gland	Chemotherapy	Survival
P10	F	40	40	Synchronously	4	4	HL-Mixed cellularity(IV)	Lymph node	Chemotherapy	Dead
P11	F	66	66	Synchronously	8	8	MM	Bone marrow	Chemotherapy	Dead
P12	F	55	55	Synchronously	14	14	NHL-TCL(III)	Spleen	Chemotherapy	Survival
P13	F	47	47	7	12	11	NHL-TCL(IV)	Lymph node	Chemotherapy	Dead
P14	F	74	76	52	26	4	NHL-	Lung	Traditional Chinese	Dead

							DLBCL(IV)		medicine	
P15	F	52	52	Synchronously	4	4	NHL-DLBCL(IV)	Lymph node	Chemotherapy	Survival
P16	F	25	38	156	-	10	AML-M5	Bone marrow	Chemotherapy	Survival
P17	F	31	48	204	-	0	Plasmacytoma	Bone marrow	Chemotherapy	Dead
P18	F	47	58	132	-	0	NHL-TCL(II)	Neck lump	Chemotherapy	Survival
P19	F	45	48	40	-	7	AML-M2	Bone marrow	Chemotherapy	Dead

*** P16-P19 were excluded from baseline analysis because of incomplete data when diagnosed with SLE. Abbreviations: SLE, systemic lupus erythematosus; HM, hematological malignancies; AML, acute myeloid leukemia; NHL, Non-Hodgkin's lymphoma; HL, Hodgkin's lymphoma; DLBCL, diffuse large B-cell lymphoma; TCL, T-cell lymphoma; MALT, mucosa-associated lymphoid tissue; MM, multiple myeloma; ASCT, autologous stem cell transplantation.**
