# Supplementary material for: Hematological malignancies in systemic lupus erythematosus: clinical characteristics, risk factors, and prognosis—a case-control study
Source: Arthritis Res Ther. 2022 Jan 3;24:5. doi: 10.1186/s13075-021-02692-8 (PMC8722144; doi:10.1186/s13075-021-02692-8)
Supplement: Supplementary file 3 — Additional file 3: Supplementary Table 3. The effects of medication exposure on HM risk in SLE patients. [file 13075_2021_2692_MOESM3_ESM.pdf]

Supplementary Table 3. The effects of medication exposure on HM risk in SLE patients

**Parameter**	**Univariate logistic analysis**	
	***OR*（95% *CI*）**	***P*-value**
Glucocorticoids	0.361 (0.055-2.388)	0.291
IVIG	0.362 (0.074-1.781)	0.211
Cyclophosphamide	-	0.999
Mycophenolate mofetil	0.308 (0.063-1.505)	0.146
Methotrexate	-	0.999
Leflunomide	-	0.999
Azathioprine	1.964 (0.166-23.249)	0.592
Cyclosporin	-	0.999
Tacrolimus	0.607 (0.067-5.469)	0.656
Thalidomide	8.615 (0.725-102.379)	0.088
Hydroxychloroquine	0.143 (0.041-0.504)	**0.002***
Rituximab	-	0.999
Belimumab	-	1.000

*** Statistical significance ($P < 0.05$). Abbreviation: IVIG, intravenous immunoglobin.**
